# Turning over New Leaves

**Published:** 1889-10-05

**Authors:** 


### Turning Over New Leaves.

(Books for Review should be sent to The Editor, The Lodge, Porchester Square, W.)

### ALGERIAN HINTS FOR TOURISTS.*

Mr. Flower, while partly an invalid, spent a winter in Algiers, and was struck by the fact that guide-books missed mentioning several points of importance. He has, therefore, from his own experience, drawn together a few very useful hints anent easy tours into the interior, the sanitation of the hotels, etc., and anyone wintering in Algiers would do well to get this little book.

* "Algerian Hints for Tourists." By Charles E. Flower. Stanford.

